# e-Book on *Closteroviridae*

**DOI:** 10.3389/fmicb.2013.00411

**Published:** 2013-12-27

**Authors:** Ricardo Flores, Pedro Moreno, Bryce Falk, Giovanni P. Martelli, William O. Dawson

**Affiliations:** ^1^Plant Stress Biology, Instituto de Biología Molecular y Celular de Plantas, Consejo Superior de Investigaciones Científicas-Universidad Politécnica de ValenciaValencia, Spain; ^2^Centro de Protección Vegetal y Biotecnología, Instituto Valenciano de Investigaciones AgrariasMoncada, Valencia, Spain; ^3^Department of Plant Pathology, University of CaliforniaDavis, CA, USA; ^4^Department of Soil, Plant and Food Sciences, University Aldo Moro of BariBari, Italy; ^5^Department of Plant Pathology, Citrus Research and Education Center, University of FloridaLake Alfred, FL, USA

**Keywords:** closterovirus, ampelovirus, crinivirus, Citrus tristeza virus, citrus tristeza

This e-Book on the virus family *Closteroviridae* provides an overview on some representative members of this family. Most articles are reviews on different fundamental and applied aspects, but a few are original contributions focused on more specific issues. Even if biased toward closteroviruses, and more explicitly toward *Citrus tristeza virus* (CTV) due to its economic relevance, this compilation altogether results an attractive blend that we hope will attract the attention of the aficionados of this fascinating virus family.

First, Dawson et al. (2013) discuss CTV-host interactions, highlighting that in contrast to movement of viruses in herbaceous plants that largely occurs through adjacent cells, CTV infection relies mainly on long-distance movement. Moreover, infection of certain citrus species requires different combinations of three CTV genes, possibly acquired by the virus to expand its host range. Regarding pathogenesis, it is unknown why the virus incites severe disease in some citrus species and not in others, but p23, a CTV-specific protein that is a suppressor of RNA silencing and a regulator of viral RNA synthesis appears to be the cause of some tristeza syndromes, particularly seedling yellows (SY).

Flores et al. (2013) describe the properties of this protein (p23), with a putative zinc-finger domain and some basic motifs, which is unique to CTV. Besides the functions mentioned above, p23: (i) elicits CTV-like symptoms when expressed ectopically as a transgene in several citrus species, (ii) enhances systemic infection and virus accumulation in p23-transgenic sour orange, and (iii) releases the virus from the phloem in some p23-transgenic citrus species. Furthermore, p23 accumulates preferentially in the nucleolus—being the first closterovirus protein with such subcellular localization—as well as in plasmodesmata.

While one of the economically relevant CTV syndromes (decline) can be managed by using resistant/tolerant rootstocks, the other (stem pitting, SP) cannot. Folimonova (2013) reports on the recent progress achieved on elucidating how cross-protection may work in the citrus/CTV pathosystem. Only isolates that belong to the same strain or genotype group of the virus (there are six) cross-protect against each other, while isolates from different strains do not. Intriguingly, the mechanism of cross-protection (or superinfection exclusion) by CTV requires a specific viral protein, p33. These findings open the door for the selection of protecting isolates.

In this same context, Lee and Keremane (2013) elaborate on the history of CTV in Florida and on the methods developed to select mild isolates that could protect against strains inducing decline of trees grafted on sour orange, a rootstock much valued for the quality of the fruit produced and for its tolerance to citrus blight, a disease of unknown etiology, and to phytophthora root rot. The final aim was to identify mild isolates that when inoculated in the existing field trees could extend their productive life and facilitate a more graduate replanting with trees propagated on tolerant rootstocks.

Also on a historical framework, Wang et al. (2013) have examined the collection of Californian CTV isolates maintained in the Citrus Clonal Protection Program (CCPP) in Riverside since 1914. Analyses of isolates from this collection using multiple molecular markers have found genotypes T36, VT, and T30 at high frequencies, with T30 and T30 + VT being the most abundant. Phylogenetic reconstructions using the CTV coat protein gene have resulted in seven clades: five associated with standard genotypes (T36, VT, T30, T3, and B165/T68) and two unrelated. Reduced phylogenetic diversity and virulence was observed in isolates collected in central California between 1957 and 2009 in comparison to those of southern California collected in early times (1957–2009). Biological characterization also indicated a reduced number and less virulent SP isolates compared to SY isolates introduced to California.

Ambrós et al. (2013) have tried to extend their CTV genetic system, which is based on agroinfiltration of *Nicotiana benthamiana* with T36-based plasmids and results in systemic infection and production of enough CTV virions to infect citrus by slash inoculation. Using oncogenic *Agrobacterium* strains they have observed induction of tumors expressing GUS in different plant species, including citrus, but systemic infection only in *N. benthamiana.* Moreover, mechanical inoculation of CTV virions to *N. benthamiana* agroinfiltrated previously with a silencing suppressor resulted in systemic infection with T36, but not T318A, which replicates in protoplast of this plant to the same extent as T36. Finally, T36 was graft-transmitted from infected to healthy *N. benthamiana* plants agroinfiltrated previously with a silencing suppressor. These data indicate that extension of this genetic system still needs considerable improvement.

Harper (2013), by examining the complete genome phylogenies of 36 CTV sequences, tackles their classification into six strains or genotypes (T36, VT, T3, RB, T68, and T30 exhibiting a wide range of phenotypic characteristics) and dissects the major evolutionary processes that led to their formation: (i) ancestral diversification of the major CTV lineages, (ii) conservation and co-evolution of the major functional domains within, though not between CTV genotypes, and (iii) extensive recombination between lineages that have given rise to new genotypes. Knowledge of the selective pressures acting upon CTV strains is crucial to the development of cross-protection programs for synthesis of CTV-based viral vectors for field release, and for breeding of new resistant citrus cultivars.

Rubio et al. (2013) deal with a similar topic, but expanded to the family *Closteroviridae.* They conclude that the major factors that have shaped the genetic structure and diversity of this family of viruses comprise: (i) a strong negative pressure that seems responsible for the high genetic stability of some viruses, (ii) human transport of infected propagative material that has caused dispersion of genetically similar virus genotypes, (iii) recombination between divergent sequence variants resulting in generation of new genotypes, (iv) interactions between virus strains or between different viruses in mixed infections that may affect the final outcome, and (v) genetic drift caused by host change or insect transmission leading to changes in the viral population.

Bar-Joseph and Mawassi (2013) focus on the finding that the molecular characterization of CTV and other members of the *Closteroviridae* has revealed that, in addition to genomic and subgenomic RNAs, infected plants often contain one or more double-stranded defective RNAs (dRNAs) of various sizes, most of which contain diverse internal deletions flanked by the two genomic termini. The roles and biological functions of dRNAs remain *terra incognita*, but one possibility is that these abundant double-stranded RNAs are used as a buffering system to protect the large and fragile viral single-stranded RNA genomes from being targeted by the RNA silencing defense of the host.

Gushchin et al. (2013) report on an intriguing observation related to replication of *Beet yellows virus* (BYV), the type species of genus *Closterovirus.* Infection by eukaryotic viruses induces formation of membranous compartments, wherein replication occurs. Specifically, complexes from cell membranes of endoplasmic reticulum (ER) or mitochondrial origin appear in closterovirus infections. Computer-assisted analysis predicts several putative membrane-binding domains in the central region (CR) of the BYV polyprotein 1a. Transient expression in *N. benthamiana* of a hydrophobic segment of the CR results in reorganization of ER into ~1-μm mobile globules, suggesting that this segment may be involved in the formation of multivesicular complexes in BYV-infected cells.

Melzer et al. (2013) have used pyrosequencing to characterize the genomes of closteroviruses infecting a single common green ti plant *(Cordyline fruticosa* L.) in Hawaii. Besides confirming the presence of *Cordyline virus 1* (CoV-1), sequence analysis has unveiled three additional closteroviruses (CoV-2 to -4), which based on the divergence of several viral proteins, represent four distinct closterovirus species. Phylogenetic reconstructions indicate that CoV-2, CoV-3, and CoV-4, together with *Little cherry virus 1* and *Grapevine leafroll-associated virus 7*, form a distinct clade within the family *Closteroviridae.*

The chapter by Dolja and Koonin (2013) summarizes advances in closterovirus research during the last several years, explores the relationships between virus biology and vector design, and outlines the most promising directions for future application of closterovirus-based vectors. These vectors offer high genetic capacity and stability, together with applicability to important woody plants such as citrus and grapevine. The description of the problems found (and their solutions) when designing vectors derived from the *Grapevine leafroll associated virus 2* is particularly illustrative.

Maree et al. (2013) provide an overview on *Grapevine leafroll-associated virus 3* (GLRaV-3), the type species for the genus *Ampelovirus*, which is regarded as the most important causative agent of grapevine leafroll disease (GLD). Complete genome sequencing of several isolates has revealed the existence of genetic variants, and characterization of the subgenomic RNAs has supplied insights into the replication strategy and the putative function of some viral proteins. Deep sequencing, apart from being a fine diagnostic tool, has furnished a more penetrating view of the complexity of viral infections and of the underlying plant pathogen interactions.

Almeida et al. (2013) discuss the ecology and management of GLD, focusing primarily on GLRaV-3, the most important virus species within the complex causing this disease. After introducing various aspects of GLD biology and ecology, the authors report on disease management case studies from four different countries and continents (South Africa, New Zealand, California-USA, and France), and end highlighting scientific gaps that must be filled for the development of knowledge-based sustainable GLD management practices.

Moving to the genus *Crinivirus* with a bipartite genome, Kiss et al. (2013) review replication and interactions with the host of the type species, *Lettuce infectious yellows virus* (LIYV). LIYV RNA1 encodes proteins involved in replication, which results in formation of vesiculated membranous structures where most likely this process occurs (see above). Four of the LIYV RNA2-encoded proteins, CP (major coat protein), CPm (minor coat protein), Hsp70h, and p59 are virion structural components, and CPm is a determinant of whitefly transmissibility. P5 is a small protein encoded at the 5′ end of RNA2 and its ortolog in BYV is localized to the ER and plays a role in cell-to-cell movement. The other small protein, p9, is unique to members of the genus *Crinivirus.*

A previous study using an AlexaFluor-based immunofluorescent localization assay has shown that retention of LIYV virions in the anterior foregut of its whitefly vector is required for virus transmission. Ng (2013), by incorporating photostable fluorescent nanocrystals, such as quantum dots (QDs), has improved the assay for the *in vitro* and *in situ* localization of LIYV virions. Immunoblot analyses resulted in a virus detection limit comparable to that of DAS-ELISA, and in membrane feeding experiments they revealed that specific virion retention in whitefly vectors corresponded with successful transmission.

Finally, the article by Tzanetakis et al. (2013) provides a detailed review on the epidemiology of the genus *Crinivirus*, most of whose members have been characterized in the last 20 years. Criniviruses have emerged as a major agricultural threat to important horticultural crops—including tomato, potato, lettuce, and cucumber—at the end of the twentieth century with the establishment and naturalization of their whitefly vectors, belonging to genera *Trialeurode*s and *Bemisia*, in temperate climates around the globe.
